# A Taxonomy of Pressure Sensors for Compression Garment Development

**DOI:** 10.3390/s25144445

**Published:** 2025-07-17

**Authors:** Gabriella Schauss, Allison P. A. Hayman

**Affiliations:** Smead Aerospace Engineering Sciences Department, University of Colorado Boulder, Boulder, CO 80309, USA; apanders@colorado.edu

**Keywords:** systemized review, on-body sensors, wearables, functional design, sensor performance

## Abstract

Recent pressure sensor research often focuses on developing sensors for impulse applications, including touch sensors, e-skin development, or physiological monitoring. However, static loading applications, such as those needed for compression garment design, are significantly under-researched in comparison. Many technology solutions do not translate across applications, as static loading requires measurements which have high accuracy, high precision, and low drift. To address the gap in sensor development between impulse and static applications, we define a literature-based taxonomy providing two conceptual classifications based on sensor functionality and specific design characteristics. The taxonomy’s utility is demonstrated through the mapping of sensors onto compression garment development phases by matching application requirements with sensor performance. The taxonomy developed will advance research and the industry by providing a roadmap of how sensor characteristics influence performance to drive a focused development for future sensors, specifically for compression garment innovation.

## 1. Introduction

Wearable pressure sensors are attracting increasing research interest. Embedding wearable objects with sensors allows for the collection, exchange, or acquisition of data without human intervention [1]. To facilitate the large-scale progression of technological integration, the design and testing of novel pressure sensors have been directed towards dynamic or impulse-based applications where high sensitivity, quick response times, and large measurement ranges are advantageous [2,3,4,5,6]. These pressure sensors have applications in areas such as touch sensors [7], electronic skin (e-skin) [8], or physiological monitoring [9]. While the focus on design for these dynamic applications has created a vibrant research field, it leaves a gap in the development of pressure sensors for static, long-duration pressure-loading applications such as medical bandages [10], compression garments [11], or novel spacesuits [12]. In these applications, sensing needs are driven by high accuracy, high precision, and low drift [13,14,15]. As such, the prevalence in the field of dynamic sensors leaves technological gaps in the development of an entire body of sensing applications.

Much of the development of compression garments has been advanced in medical, space, athletic, and recreational industries [11,16,17,18,19,20]. Currently, within these fields, pressure applied from compression garments often goes unreported or is not empirically verified, especially in studies for sports or athletics [11]. Additionally, measurements that are reported by researchers often have high uncertainty associated with them due to measurement errors within the pressure sensors. For example, within the medical field, evidence from clinical studies on compression stockings for the prevention of deep vein thrombosis found that measured pressure was often significantly different from the expected value [21]. Additionally, claims about the effects of graduated compression on athletic performance are often inconsistent across studies [11]. These inconsistencies have often been attributed to differences in body types across subjects as well as inadequately applied pressure due to errors in the pressure sensors used during verification [11,22]. However, the presence of multiple error sources makes it difficult to delineate the contribution of magnitude, distribution, or coverage of pressure garments on study outcomes [11,15]. The high uncertainty in the reported pressures and heterogeneity across studies results in a lack of fundamental understanding of the interface between the compression garment and the body. Information about the applied pressure is essential for subsequent efforts to compare findings across studies as well as provide a more comprehensive understanding of the effect of certain applied pressures on the body [11]. Overall, this lack of a reliable measurement system has resulted in a stagnation in the development of high-performance compression garments across all industries. While research on impulse pressure sensors is ever-growing, there is currently an inability to understand the capability of those pressure-sensing technologies as they translate across applications.

## 2. Materials and Methods

**Figure 1:**
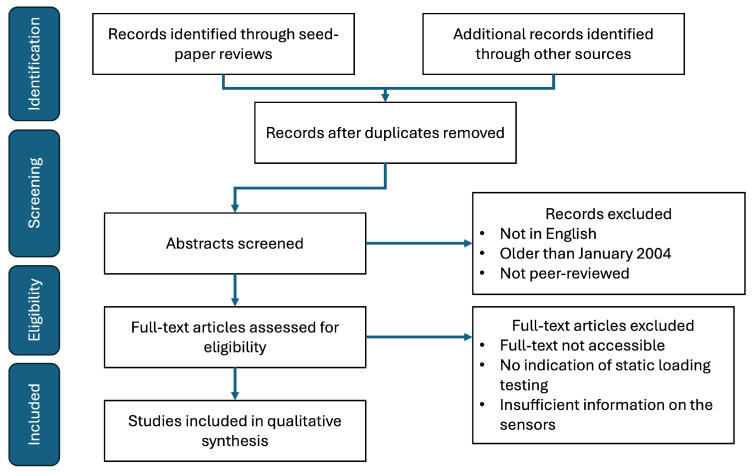
PRISMA flow diagram illustrating paper selection [23].

To map the compression garment requirements to specific pressure sensor technologies to create the taxonomy, a systemized review of the literature using a modified PRISMA flow was conducted. Publications were identified through a seed-driven paper review [23]. Figure 1 shows the process in detail. Due to the multidisciplinary nature of pressure sensor research, the literature included in this review was gathered from multiple outlets and various disciplines. The literature was grouped, analyzed, and discussed from the perspective and needs of compression garment developers and other compression garment designers. In doing so, the literature can be leveraged to identify sensor elements that may allow for an optimization of critical sensor performance metrics.

As a first step, a manual keyword search in Google Scholar, ACM conference proceedings and IEEE conference online databases was performed. Journals and conference proceedings from the fields of textile sensors, electrical engineering, advanced materials, and medicine were also searched. The search keywords were ‘pressure sensors’, ‘compression garment’, and ‘skin interface’. This initial search resulted in a collection of seed publications about wearable pressure sensors, measurement methods for compression garments, and on-body testing methodologies. After identifying a publication as relevant for the review, its citations and references were searched to find further relevant papers. This process was repeated for all relevant papers until no additional papers relevant to pressure sensors for compression garments could be found.

Defined exclusion criteria were divided into two categories: metadata and technical. For metadata criteria, studies were excluded if the publication was not a full-text research article or conference paper, not peer-reviewed, not in English or not published in an English-language journal or conference, or was not published within the last 20 years (i.e., papers had to be published after 2004). This latter criterion was established to acknowledge the rapid advancements in sensor technologies within this timeframe.

For technical criteria, originally, all publications that did not include testing specific to on-body compression garments were excluded. This strict exclusion criterion resulted in only five papers [10,24,25,26,27]. As such, the exclusion criteria were revised so that publications were excluded only if they did not test or report at a pressure range that was applicable for compression garments; publications did not have testing or developmental considerations for static loading; or publications did not report on the materials or fabrication methods of the sensors. The technical criteria intended to omit publications that only addressed impulse-specific applications such as touch sensors [6] or gait analysis [28]. These criteria allowed us to create a focused overview of sensors related to compression garments and provided a rich dataset to derive a taxonomy. It should be noted that while system-specific design choices were not incorporated into the taxonomy, they were often important aspects in the down selection of included papers as they provided information on the operational relevance of the testing. Overall, this process resulted in 26 publications being incorporated into the taxonomy.

All qualifying literature was organized by publication metadata, sensor design characteristics, and performance metrics of each sensor developed or tested within the study. Beyond mapping the sensor literature to the taxonomy, information about the data collection, testing methods, metrics reported, analysis, and findings was collected. Based on the literature analysis, the taxonomy was constructed in two main parts. The first part addresses the sensing mechanism of the sensor. The second part of the taxonomy addresses the sensor’s fabricated form indicated by specific design attributes such as materials and structures. Through an iterative process, the taxonomy was adapted and reorganized into tiers to group the sensor’s performance in a way that reflects functional design choices and advantageous sensor metrics.

## 3. Results

The sensors analyzed represented a diverse set of designs and applications. A total of three different application areas were identified with four publications not calling out a specific application. The medical and HCI fields were predominant in research output, with ten publications for each industry. This was expected as there is extensive existing research on bed sores [29], compression garments [30], and prosthetic monitoring [31]. Additionally, most wearable pressure sensor publications reside within the more generalized research realm of HCI. Only one publication specifically identified sports as its intended application industry. This was most surprising as there is a heavy interest in and use of compression garments for athletic performance and recovery. A breakdown of eligible publications by industry is given in Table 1.

### 3.1. Pressure Sensor Definitions

Identifying critical sensor metrics and defining the application space is needed to create a taxonomy. The analysis was started by providing definitions of sensor metrics and characteristics, which establishes consistent language for the taxonomy. A metric is defined as a system or standard of measurement, while a characteristic is defined as a non-measurable aspect of a sensor’s design. From the literature, primary sensor metrics that were considered critical in determining performance and applicability to MCP applications were identified. The review found that many terms associated with sensor design, testing, and performance were used inconsistently. Consequently, a description and definitions of primary sensor performance metrics and characteristics in this section are provided to establish standardized language.

*Static and Dynamic Loading*: Static and dynamic loading refer to the difference in the time duration that pressure is applied to a sensor. Within the literature, the defined time durations for static loading can be highly variable between studies, ranging from seconds to hours [10,26,46]. These durations and pressure magnitudes are often defined by an intended application. In contrast, dynamic loading or cycling testing refers to pressure that begins and ends rapidly. The two loading methods can be differentiated through compressive impact (dynamic loading) and compression (static loading) [34]. For the development of the taxonomy, papers must include a period of static loading. While the ideal static loading testing would span hours, as the sensor might be used in an operational setting, for increased inclusivity, studies of shorter loading durations were included. As such, static or quasi-static loading incorporated studies that loaded sensors for more than 5 s. This duration, while short, allowed for the exclusion of sensors that were only tested for impulse-based performance.

*Accuracy*: Accuracy refers to how closely the measured value is to the actual value. It is often reported as the difference between the two values expressed as a percentage [49]. Accuracy can be affected over time by several different factors, including material creep, measurement drift, or motion artifact. Accuracy may be measured and reported for a single trial, between multiple trials (repeatability), or between trials of different sensors (generalization) [50]. Calibration can be used to increase the accuracy of measurements by accounting for environmental conditions, material creep, or hysteresis.

*Linearity*: Linearity is a measure of how proportional the measurement is related to a range of stimuli [50]. It is calculated by comparing the sensors’ output data against the input parameter. Linearity is often expressed as the maximum difference between measurement points and a linear regression line. Increased linearity is often advantageous since it allows for easier calibration and minimizes uncertainty in the output scaling [49,51,52].

*Hysteresis*: Hysteresis is the extent to which sensor response depends on the time loading or unloading history. Purely elastic materials possess identical loading and unloading profiles, but viscoelastic materials have internal energy dissipative mechanisms that produce different loading and unloading paths. Hysteresis is measured as the energy loss in a system, which is calculated by taking the difference between the applied control signal and the resulting change in the system [50]. Large hysteresis reduces both measurement accuracy and repeatability of a sensor [53].

*Sensitivity and Resolution*: Sensitivity is a term that describe how a sensor responds to a signal and quantify measurement specificity. Sensor sensitivity refers to the change in output per unit of input pressure variation. The sensitivity of pressure sensors can be affected by factors including temperature changes or mechanical stress, and is often traded off with linearity, as sensors with high sensitivity usually have a small linear sensing range [50].

*Drift*: Drift is the deviation of the sensor response over time and is relevant for measurement accuracy, especially for static loading applications [50]. For polymetric materials, drift often originates from restoration changes due to viscoelastic properties that are intrinsic to the material. Environmental factors such as changes in temperature or humidity may result in measurement drift [54].

*Reliability*: Reliability is the likelihood that a sensor will perform its intended function without failure or degradation over a specific period and under specific conditions [55]. Reliability is most critical for operational settings where sensor functions need to be performed during their time of use. However, a reliability measurement does not need to span the entire operational time and can be on demand, as in the case of an impulse signal, or prolonged, as applied static loading [55]. Reliability is reported in cycle testing, which provides insight into the sensors’ degradation with repeated loading.

Sensor metrics were extracted from the included studies. It should be noted that not all of the papers provide information on each metric identified, as testing and evaluation criteria differ between studies. Table 2 provides a summary of the sensor metric.

### 3.2. Functional Form: Sensing Mechanism

Within general wearable pressure sensor reviews, five primary sensing mechanisms are often identified: piezoresistive, capacitive, piezoelectric, triboelectric, and pneumatic [57]. After filtering, parsing, and grouping pressure sensors specific for compression garment applications from the down-selected publication repository, only three sensing mechanisms were found in the remaining papers: piezoresistive, capacitive, and pneumatic. It should be noted that triboelectric and piezoelectric sensing mechanisms, which are often cited in the literature as effective pressure sensors, were retained [58,59,60] because those pressure sensors are limited in measuring static pressure due to their dynamic mechanisms, such as friction, which is used to generate an electrical signal. Additionally, these sensing mechanisms still require improvements in reporting accurate measurement values over duration loading [58,61,62,63]. Of the three remaining mechanisms, piezoresistors were divided into strain gauges, microfluid channels, contact resistors, and electrochemical to increase the granularity of the Functional grouping. For capacitive and pneumatic sensors, the number of included publications for each of the functional forms did not allow for the discretizations of the sensing mechanism. Publications organized by sensing mechanism are given in Table 3.

*Piezoresistive*: Generally, piezoresistive sensors have a thin profile that is advantageous for integration into a variety of applications [4,64]. They also have been shown to have high detection, short response times, large detection ranges, and low power consumption [4,57]. However, they can be influenced by factors such as temperature in long-term mechanical stress which reduces their accuracy and requires proper, individualized calibration throughout their use [65,66]. They are the most extensively developed and studied sensing mechanisms due to their simple designs and manufacturing processes and have also been used in commercial applications. Within the 26 publications in the taxonomy, 20 of them reported or developed piezoresistive sensors.

- **train Gauge**: Solid metals are desirable materials for resistive pressure sensors because of their hysteresis-free properties [67] and fast response time in the elastic region [7]. As pressure is applied to a strain gauge, deformation leads to changes in length and cross-sectional area, resulting in a change in resistance.
- **Microfluidic Channels**: Microfluidic channels are often filled with liquid metal, such as eutectic gallium–indium (EGaIn), and are embedded in an elastomer to detect normal pressure. Challenges with liquid metals lie in limitations in reducing the size and the high-pressure regime [32,35,68].
- **Contact resistance**: This sensing mechanism relies on two resistive surfaces encased in an insulating matrix that make contact as pressure is applied. Disadvantages of this mechanism are drift, hysteresis, and slow response due to the viscoelastic effects of insulating soft elastomers [40].
- **Electrochemical**: When a mechanical force is applied to the sensor systems, an electrolyte within causes a chemical reaction forming an electrochemical cell [46,69]. This reaction emits an electrical signal proportional to the applied stimulus. These sensing mechanisms are often self-powered, which is highly advantageous for wearable applications [38].

*Capacitive*: Capacitive pressure sensors consist of two conductive parallel electrodes that are separated by a medium. Changing the separation of the two parallel plates is the most used technique for quantifying pressure changes [70]. Variance in the materials selected for the dielectric and electrode layers can tune the capacitance and resulting output signal. Capacitive sensors are often reported to have high sensitivity, fast response, and wide dynamic range [44,45,70,71]. Primary limitations associated with capacitive sensors are the separation distance between electrodes, parasitic effects from motion artifact, and the drift in capacitance [71,72].

*Pneumatic*: Pneumatic deformation sensors are soft bladders, and when deformed, the air pressure inside the bladder changes and corresponds to the value of applied pressure. In the commercial sector, this method of measuring on-body compression is one of the most common, especially in the medical field [11,14,22,29]. The primary limitation of these sensors for all compression garment applications is the area and thickness of the sensors. While effective for regions of the body with large radii of curvature (legs and upper arm) they are not suitable for smaller regions such as hands. For medical applications pneumatic compression devices are often used as therapeutic strategies for lymphatic drainage [30]. For these applications pressure on the body can be accurately measured through the gauge pressure within the pneumatic device. This is an effective method of compression application but is not able to be translated into other applications such as athletics or space environments, where users have more complex operational requirements [73,74].

**Table 3:** Eligible publications organized by each sensing mechanism.

Sensing Mechanism		Eligible Publications
Piezoresistive	Strain Gauge	[10,24,26,27,31,34,35,36,39,42,46,47,48]
	Microchannels	[32,33,35]
	Contact	[40,43,75]
	Electrochemical	[38]
Capacitive		[9,37,43,44,45,48]
Pneumatic		[22,25,27]

### 3.3. Fabricated Form: Sensing Structures and Materials

The fabricated form of the sensors looks at specific structural and material properties. These structures and materials may, but do not always, correspond to a sensing mechanism. As such, exploring and categorizing sensors through their structures and materials provides an alternative view of sensor design and a more holistic understanding of how sensor characteristics can relate to their performance. Publications that address fabricated form are given in Table 4. Moreover, 3D buckling structures offer a higher degree of deformation resulting in higher sensitivity to normal pressure. These structures have been utilized for piezoelectric and resistive sensors. The development of 3D buckling structures keeps the advantages of conventional metal strain gauges such as linear response, reliability, and simple fabrication process while improving sensing range and sensitivity [26].

- **3D Geometry**: 3D geometry converts pressure into bending deformation inducing changes in the resistance of the strain gauge. Adapting a 2D planar structure into a 3D geometry increases the sensor response under pressure [26,56].
- **Conductive Yarns**: Yarns are often used in traditional textile fabrication methods to create 3D structures through knitting, weaving, and embroidering. In doing so, sensor sensitivity or sensing range can be adapted [42,46].

*Microtexturing*: Flat thin films are often susceptible to viscoelastic creep. The addition of microstructures on a surface allows for higher stability during elastic deformation resulting in faster response times and a reduction in drift [53,76]. Additionally, micropores reduce the effective modulus and enhance the sensitivity of the sensor. Overall, a diversity of surface geometry enables sensors to be tailored both to sensitivity and pressure ranges.

- **Nanowires and Ribbons**: Nanowires are broadly defined as structures that have diameters of tens of nanometers or less and are commonly found as carbon nanotubes. Nanoribbons have microstructures that allow them to stretch with dynamic movement and provide high spatial–temporal sensitivity and reliability [38,77].
- **Conductive Surface Alterations**: Deposition of conductive materials on a surface is one of the more common methods of sensor development and is used in both capacitive and piezoresistive sensors. The conductive materials are often nickel, gold, or aluminum, which create low-profile conductive surfaces [26]. Graphene or other forms of conductive carbon are also increasing in the development of pressure sensors [40].

*Sponges and Foams*: Textile-inspired fiber/mesh pressure sensors enhance mechanical flexibility due to their thin form factor, high fracture point, low-cost production, and easy integration [36]. Base materials have significant implications on the stability and reliability of the sensor as well as considerations for biocompatibility.

- **Dielectric Composites**: There are many types of dielectric composites, including polydimethylsiloxane (PDMS) and dielectric electroactive polymers (DEAP) [44,45,47,78]. PDMS is the most explored elastomer for dielectric composites due to its high elasticity and biocompatibility. PDMS is also used as structural substrates to increase measurement stability or sensor reliability.
- **Poly(3,4-ethylenedioxythiophene) (PEDOT)**: PEDOT is an aqueous conductive, biocompatible polymer that has recently been used extensively in electronic textiles and flexible electronics [64,79]. PEDOT has been shown to be effective as a conductive element through doping non-woven or foam structures creating textile-based electrodes or complex surface structures [9,39,42].
- **Graphene Coating**: There are multiple different forms of graphene for pressure sensor applications, but reduced graphene oxide (rGO) has become one of the most common types. rGO refers to graphene-like nanosheets that can be synthesized via chemical reaction, electrochemical reduction, or thermal reduction [80,81]. These conductive sheets are often embedded or used to coat insulative substrates [36,47].
- **Electrolytes**: The incorporation of electrolytes into pressure sensors is a new area of research. Electrolytic materials have many of the advantages of triboelectric or piezoelectric sensors, including being self-powered, while still being able to effectively measure static pressure [38,82].

*Fluidics*: Through the use of fluidic structures, mechanical deformation can be transformed through liquids or gases providing a method of accurate pressure measurement. Liquid metals or ink are often commonly used to develop sensors as there is a stable relationship between the electric resistance and applied pressure [32,83]. Gaseous or pneumatic systems may also be categorized as fluidic as the pressure applied to a pneumatic system results in a deformation or displacement of the gas inside of it, providing a measurement [22].

- **Conductive Inks and Alloys**: Conductive inks are very common, especially in commercially available pressure sensors. One conductive ink is EGaIn, which is characterized by high surface tension, low toxicity, and low viscosity, making it a popular choice for microfluidic applications [10,24,32].
- **Bladders**: Bladder structures are most commonly filled with gas and have been shown to have good repeatability and are inherently insensitive to shear force. They are widely used in measuring the skin–garment interface and commercially available [22,27,84].

## 4. Discussion

**Figure 2:**
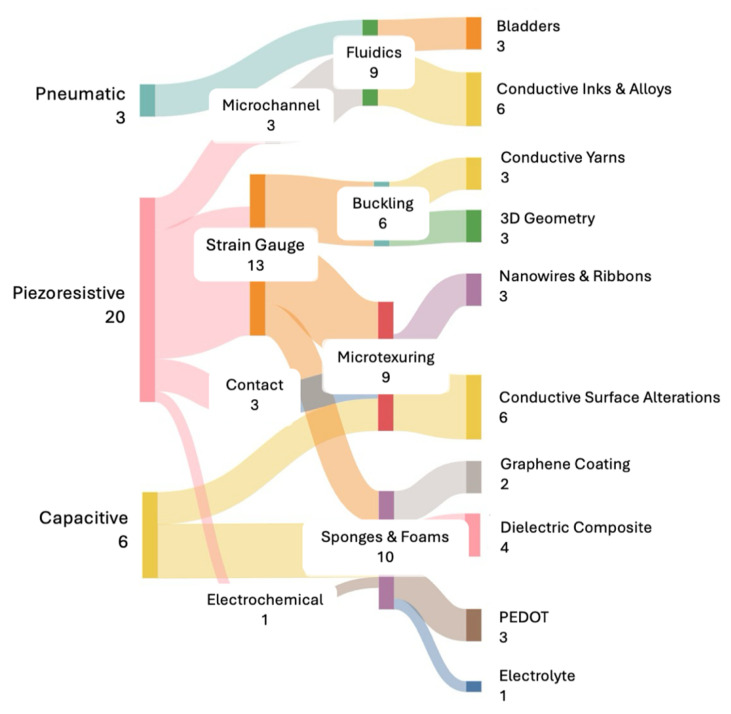
Sankey diagram illustrating flows of three components involved in sensor performance generated from an analysis of the included publications. The thickness of the lines is proportional to the number of relevant publications for each connection [85].

The goal of the taxonomy was to allow for improved road mapping of the relationship between sensor characteristics and sensor performance to further the development of pressure sensors for specific compression garment applications. The developed taxonomy provides a framework for how to proceed from general sensor characteristics to specific sensor performance through multiple tiers. Figure 2 provides a Sankey diagram illustrating the relationship between the sensing mechanisms and fabricated forms of the sensors. The complexity of this taxonomy is the organization of the properties of the sensors themselves as well as their performance properties. These two aspects increase complexity by creating a multiple-dimensional dataset that has dynamic interactions within and across taxonomy tiers. Additionally, the literature does not uniformly report fabrication or performance. Therefore, gaps in performance metrics may create gaps in the taxonomy which provides areas for debate.

In the analysis of publications, it was found that many performance metrics and testing methods are often inconsistent across the literature. As a result, the translation of findings from one application to another is increasingly difficult, barring the potential utilization of technological advancements across industries. A large body of work addressing the need for pressure sensors without explicitly using or testing them in static loading conditions was found. Because of this, those publications did not meet the inclusion criteria and were not used in the results. However, this indicates that there are publications that may address the needs of compression garment applications but currently do not undergo the testing required to draw definitive conclusions about their potential performance for these applications. The focus on pressure sensors for measuring body compression from elastic garments is due to the research background of the authors. The taxonomy is a useful tool for HCI as the advancement in this field further propagates wearable systems and wearable electronics in the future. Table 5 provides an overview of each sensing mechanism, specific sensor requirement, and sensor mapping based off the current taxonomy.

### 4.1. Sensors for the Understanding of Pressure Physiology

Understanding the fundamentals of pressure physiology is critical to medical, athletics, and sports applications. Sensors for physiological understanding require high sensitivity, low drift, high accuracy, high resolution, and high linearity. Past work often used pneumatic sensors for medical applications, but these sensors have relatively large profiles and surface areas which limits them to large areas of the body and makes them susceptible to the effects of body curvature. From the taxonomy, sensors that exhibit metrics that align with physiological requirements are piezoresistive strain gauges that use 3D frameworks to leverage buckling in response to external pressure. Sensors with these characteristics reported negligible drift, high linearity (>0.99), high sensitivity, and negligible effects from curvature [26,56]. In addition, sensors that use PDMS as a base substrate discuss the ability to customize the modulus of the PDMS allowing for the fine-tuning of the pressure detection value, pressure sensitivity, and pressure detection range, all of which are key metrics in physiological applications [26]. These findings are promising in the development of pressure sensors to advance the fundamental understanding of compression physiology.

### 4.2. Sensors for Facilitating Design and Development

The design and development phase has the least number of requirements for sensors. Generally, sensors for compression garment design and development must have high resolution, high repeatability, and high reliability. These requirements ensure the pressure sensor allows a designer to compare across designs to implement materials, structures, or technology, which enables compression garment development across all fields. Force sensing resistors (FSRs), which can be categorized as piezoresistive strain gauges with conductive inks, have long been used as a method to measure on-body pressure during the design phase as they are low-cost and readily available. This comes at the cost of low repeatability [10,24,27]. While repeatability is a critical requirement defined by the design and development application, it may be that in the initial stages of development, a decrease in repeatability trades favorably for the accessibility of these sensors. Alternatively, piezoresistive microchannel sensors, which also use conductive ink or alloys, show promise in providing pressure measurements with high resolution, high repeatability, and reliability (stable over 1000 cycles) [32,56]. Additional research will need to be conducted to decrease their size so that they can be applied to areas of the body with small radii of curvature, such as the hand.

### 4.3. Sensors for Performance Verification of Pressure Systems

Compression garment system verification may be the most difficult application for sensor development due to the need for high measurement certainty and accuracy when compared to other application phases. Being able to distinguish between deviations in measurements via compression garment degradation or pressure sensor errors has been a source of uncertainty in the past, decreasing confidence in both the sensors and compression garments. Sensors for system verification require high sensitivity, accuracy, linearity, repeatability, and reliability. The most difficult requirement for test sensors is repeatable accuracy over long periods of static loading. While there may be sensors that do excel in this metric, very few studies report static loading testing with the rigor that is needed to conclude the effectiveness of those sensors for the system verification application. Within the current body of literature, the most promising sensor candidates are piezoresistive strain gauges with 3D buckling geometries [26], piezoresistive microchannels with conductive alloys [32], or piezoresistive strain gauges with PEDOT sponge-like membranes [39]. Capacitive sensors within the literature have a high amount of parasitic noise which makes measurement accuracy difficult [44,45,72]. The gap in information for this sensor application appears to be due to a lack of testing rather than the quality of the sensors developed. As such, more testing should be carried out that targets metrics for static loading applications.

### 4.4. Sensors for Enabling Operational Monitoring

Operational monitoring is unique in that it is the only one of the four applications that is used outside of the laboratory environment. Pressure sensors for operational monitoring require low drift, high accuracy, high repeatability, and high reliability to ensure users have adequate, and in some cases critical, pressure applied to the body. Being outside of the laboratory environment means that there are additional considerations for pressure monitoring including motion artifacts and a decreased signal-to-noise ratio (SNR). While not explicitly explored within this taxonomy, they are important considerations for future development. A mixed sensing mechanism design would be advantageous for operational monitoring to provide pathways for static and impulse-based input measurement [48]. For example, a piezoresistive strain gauge system may be effective in measuring static pressure from the compressive garment, while a capacitive system may be able to measure impulse loading from the environment (such as picking up an object or bumping into a wall). In this way, motion artifacts or noise from the environment can be effectively filtered out or accounted for through the dual-sensing modalities. Other alternative sensor systems, such as electrochemical sensors, provide promising initial results for operational monitoring as they are self-powered but lack the accuracy needed for applications [38]. Targeted development in this research area may elevate sensor performance to a level to meet the operational requirements of compression garments in the future.

### 4.5. Discussion

Sensor researchers and practitioners in the medical, sports, HCI, and space domains, will benefit from a clear and structured definition of sensor performance metrics as well as a pathway for targeted sensor development in the future. An ideal outcome for the taxonomy could be the following: researchers and practitioners may adjust their research endeavors or intended sensor designs in future publications according to this taxonomy. Furthermore, this taxonomy draws attention to the significant imbalance between pressure sensor development for dynamic applications in comparison to static applications. In this way, with a clear directive for future development, there will be an increase in advancement for this research space incorporating a multitude of disciplines including textile designers, material scientists, and electrical engineers.

There have been trends in the literature to explore the tactile and pain receptors in the skin to drive research development. For example, ref.[42] used a mixture of aqueous PEDOT solution and the traditional fabrication method of embroidery to create a pixelated nanowire sensing fabric inspired by Merkel cells [42,48]. Additionally, ref.[77] took inspiration from the 3D, hierarchical structures in biological systems to develop highly sensitive and responsive sensors [77]. Continued understanding and investigation into the sensing mechanisms and structural elements of biological structure to maximize functionality may result in many developments in both static and dynamic sensing modalities.

One of the primary challenges in developing the taxonomy was the interpretation and organization of reported results. A lot of the difficulty in comparing sensors or analyzing findings is due to inconsistencies in testing methods and reported test metrics. This is especially true for aspects of sensor characterization for static loading. Static loading in literature has a range of definitions and time durations which can range anywhere from 3 min to hours. All the sensors included in this taxonomy have textile-based components or are integrated into textile systems. Textiles have time-dependent properties, predominantly viscoelastic properties, which may have effects on pressure measurements. Viscoelastic materials have both viscous and elastic characteristics and, as such, exhibit time-dependent strain. The lack of static pressure testing significantly decreased the number of publications incorporated in the taxonomy and ultimately limits the understanding of the sensor properties. Since impulse-based applications dominate the pressure sensor development space, testing and evaluation in the literature reflect the needs of those applications leaving a gap and inability for sensors to be applied across industries.

Additionally, application or operational testing is limited. For example, sensors for on-body sensing applications are not necessarily evaluated on substrates that reflect the modulus of the skin or on surfaces that reflect the curvature of different areas of the body. The gap in testing between benchtop evaluation and operational application may result in a high error rate or produce unanticipated results. The reported performance of a specific sensor type, such as an FSR, is highly inconsistent across studies. This inconsistency is especially evident for commercially available sensors. Results are often at odds within the literature and with the manufacturer specifications [10,24,32]. Thus, additional testing on substrates relevant to the intended application is necessary when reporting on sensor performance. Additionally, while many of the papers noted sensors were constructed out of biocompatible materials, very few studies reported human skin testing for long-term comfort. For compression garment applications, comfort and biocompatibility are two critical characteristics that must be tested for.

There are whole areas of sensing mechanisms, materials, and novel structures that may apply to the development of pressure sensors for compression garments and static pressure measurement that were not incorporated into this taxonomy since the current literature does not provide an in-depth analysis of testing for static pressure applications. Thus, there may be sensors that did not meet the criteria for inclusion so not captured in this analysis. Additionally, with the increasing development of e-skin, many of the technologies have already been developed, but they may just need to be modified for a static pressure-loading application. Hopefully, the categorization and identification of these sensors provide a means for textile design, electrical engineering, material scientists, or medical professionals to explore and expand the development space.

The taxonomy should continue to evolve as additional research progresses. The defined exclusion criteria were intended to create a collection of publications that have a rich data set for sensor-type evaluation while still maintaining specificity for the intended application. In this way, informed discussion and direction can be developed but not so generalized as to no longer be directly applicable. Many sensors within the literature may be effective technologies for static pressure sensors, but due to the lack of static loading-specific testing, their performance is not well characterized in this area. This is a substantial research gap and sheds light on the focus of the developing field on impulse-based, dynamic, touch sensors.

From the included literature, categories of sensors were established. However, in some cases, categorization may be debated or reorganized into different subsets. For example, in this taxonomy, bladders were categorized as a subset of fluidics, but one could make an argument that they could also be a subset of buckling structures instead. Thus, the taxonomy’s utility is scoped at a useful granularity to apply to compressive garments but may be adapted as additional literature is published.

### 4.6. Limitations and Future Work

While this work focuses on categorizing static pressure sensors, this work can be extended in the more generalized category of pressure sensors. As such, all other pressure sensors (i.e., pressure sensors for impulse or dynamic loading) could also be clustered, grouped, and organized, as was carried out with static loading sensors. Since dynamic pressure sensors produce a larger publication set, it is possible to add them in the future. Additionally, through the development of application-specific requirements, the inclusion criteria for the development of the taxonomy can be altered for the application, expanding the use of the taxonomy beyond sensors for on-body compression garments.

As previously mentioned, a limitation of this work is a clear research gap in the development of pressure sensors for on-body compression garments. The 26 publications, even after the exclusion criteria, were relaxed to broaden the range of publications. Additionally, many of these publications used or revolved around commercially available sensors, such as FlexiForce (Tekskan) or PicoPress. Although they have a larger body of supportive literature, they have still been shown to have high variability and are prone to measurement error, making it difficult to isolate characteristics that may be advantageous for future development from those that may not be [10,27,86].

Additionally, as shown in Table 2, there is no standardized or complete testing for all the sensors, leaving gaps in the characterization of the research. Furthermore, almost none of the studies conducted testing that would be indicative of sensor degradation over long-term use, which may reflect performance in operational settings. Sensors often undergo cycle testing as a measurement of robustness and performance degradation, but this testing regime focuses on impulse-based loading for long-duration static loading. As such, to increase our understanding of how these sensors may perform in operational settings, additional sensor degradation testing needs to be included.

## 5. Conclusions

In this paper, a literature-derived taxonomy was developed to provide a method to make connections between sensor design characteristics and sensor performance metrics. The 26 publications were analyzed, and it was found that there is a need for the targeted development of pressure sensors in measuring pressure for compression garments incorporating specific design requirements. The taxonomy provides a framework for future developers allowing sensors to be identified that are well-suited for a specific set of requirements. This work establishes a common understanding of how a sensor’s design characteristics relate to specific performance metrics. In doing so, two subsets of sensor categorizations were identified: sensing mechanisms and fabricated form. Overall, this work highlights the need for targeted sensor development. Many of the promising and novel technologies used for e-skin and other prominent compression garment applications could not be incorporated into this taxonomy. This work aims to support future compression garment designers in a wide range of applications including textile fabrication, fashion and aesthetics, tactile psychology, and wearable electronics.

## Figures and Tables

**Table 1:** Number of eligible publications by industry or intended application area.

Pressure Sensor Application	Eligible Publications
Medical	[9,10,22,24,25,26,27,32,33,34,35]
HCI	[31,35,36,37,38,39,40,41,42,43]
Sports and Athletics	[44]
No reported application	[45,46,47,48]
Total	26

**Table 2:** Sensor metrics from studies included in the taxonomy.

Reference	Sensing Mechanism	Structure	Loading	Measurement Range [kPa]	Linearity [R^2^]	Hysteresis	Sensitivity [kPa^−1^]	Drift	Accuracy	Cycle Testing
[26]	Piezoresistive Strain Gauge	3D Buckling	On-body testing for up to 5 s	0–180	>0.99	<0.02%	2.2–3.1 × 10^−5^	Negligible		1000 cycles (mean delta 0.37%, SD 0.07%)
[45]	Capacitive	Sponges & Foams Dielectric Materials	Loading of 30 s	0–13	Good	Present but small	0.001		0.18 pF	400 Loading cycles
[10]	Piezoresistive, Strain Gauge	Conductive ink	5 kPa for 20 min	0–18	0.989	<10% below 10 kPa; <20% above 10 kPa		<5%		15% FSD
[33]	Piezoresistive Strain Gauge Microchannel	Buckling 3D Geometry	20 s duration	0–10		Negligible				
[24]	Piezoresistive Strain Gauge	Conductive Ink	On a subject under a compression band for 25 s	0–30						
[47]	Piezoresistive Strain Gauge	Sponges & Foams Graphene	Quasi-static compression test	0–70	Non-linear monotonic	Presence of hysteresis loops	0.23	Negligible		
[41]	Piezoresistive Strain Gauge	Microtexturing Conductive Surface	Pressure sensor array for static pressure mapping	0–25	0.998		1.2			Stable over 1000 cycles
[43]	Capacitive	Sponges & Foams	Loading of 15 s	0–14	0.978		1450			Stable over 1000 cycles
[22]	Pneumatic	Fluidics, Bladder								
[42]	Piezoresistive Strain Gauge Capacitive	Sponges & Foams PEDOT, Buckling Conductive Yarns	Consistent loading up to 350 kPa	0.2–400	Linear response from 0.2–400 kPa		8.59			
[56]	Piezoresistive Strain Gauge	Buckling 3D Geometry		0–13	0.992	Negilible				Stable over 10,000 cycles at 120 kPa
[38]	Piezoresistive Electrochemical	Sponges & Foams Electrolyte	Static sensing of approximately 90 kPa was applied	0–110		13%	10			500 cycles at 10 kPa
[40]	Piezoresistive Strain Gauge	Sponges & Foams Dielectric Materials		3.5–15			14.4			Stable over 1000 cycles
[32]	Piezoresistive Microchannel	Fluidics Conductive Inks	Current signal from static hand gestures	0-60	0.999		0.0835			
[37]	Capacitive	Sponges & Foams Dielectric Composite	10 s loading between 10–300 kPa	0–400	Nonlinear	Low	0.18			10 cycles at 450 kPa
[39]	Piezoresistive Strain Gauge Contact	Sponges & Foams PEDOT, Microtexturing Conductive Surface	Loading over 1, 3, 5 kPa	0–30			13.5			Stable over 10,000 cycles
[9]	Capacitive	Sponges & Foams PEDOT Dielectric Composite	Lower limit for 15 s	0–400	0.995	Negligible	0.51			Stable over 10,000 cycles at 167 kPa
[44]	Capacitive	Sponges & Foams PEDOT, Conductive Ink	5 s loading at 100 kPa	0–1000		7.8%	0.3			
[36]	Piezoresistive	Sponges & Foams, Graphene Coating Nanowire	15 s durations	0–27	0.99 (within 0–3.24 kPa)	Negligible	0.042–0.152			Stable over 9000 cycles
[27]	Pneumatic	Fluidics Bladder	Consecutive loading and unloading periods							
	Pneumatic	Fluidics Bladder	Consecutive loading and unloading periods	0–27						
	Piezoresistive Strain Gauge	Fluidics Conductive Ink	Consecutive loading and unloading periods	0–18						
[35]	Piezoresistive Strain Gauge	3D Buckling	20-second durations	0–13	0.992	Negligible				Negligible variations in response over 1000 cycles under pressures of 50 mmHg
[48]	Piezoresistive Capacitive	Buckling 3D Geometry Sponges & Foams Dielectric composites	Prolonged loading	0–200	Linear	7.10%	14.117			Negligible variations over 4000 cycles
[31]	Piezoresistive Strain Gauge	Microtexturing Nanoribbons				Negligible				
[25]	Pneumatic	Fluidics Bladder	Static pressures on a mannequin leg	0-13	0.99				0.4 kPa	
	Pneumatic	Fluidics, Bladder		0–27	0.99					0.4 kPa
	Pneumatic	Fluidics, Bladder		0–40	0.99					0.13 kPa
[46]	Piezoresistive Strain Gauge	Buckling Conductive Yarns	Samples were stretched and held for 3 min		0.9825					
[34]	Piezoresistive Strain Gauge	Fluidics Conductive Ink	8 h static loading	2.5–9	Linear	5%	Negligible	94%	stable over 10 loading cycles	

**Table 4:** Eligible publications organized by fabricated form, including structure and materials.

Fabricated Form		Eligible Publications
Buckling	3D Geometry	[26,33,56]
	Conductive Yarns	[42,46,48]
Microtexturing	Nanowires and Ribbons	[31,36,43]
	Conductive Surfaces Alterations	[26,40,41,44,45,56]
Sponges and Foams	Dielectric Composites	[9,37,44,45]
	PEDOT	[9,39,42]
	Graphene Coating	[36,47]
	Electrolytes	[38]
Fluidics	Conductive Inks and Alloys	[10,24,27,32,34,35]
	Bladders	[22,25,27]

**Table 5:** Sensormapping onto compression garment development phases based on critical requirements and taxonomy categorization.

Compression Garment Development Phase	Critical Requirements	Taxonomy Mapping
**Physiology**	High Sensitivity Low Drift High Accuracy High Resolution High Linearity	Piezoresistive∼strain gauges Buckling∼3D geometry
**Design**	High Resolution High Repeatability High Reliability	Piezoresistive∼strain gauges Fluidic∼Conductive Inks Piezoresistive∼Microchannels Fluidic∼Conductive Inks
**System Verification**	High Sensitivity High Accuracy High Linearity High Repeatability High Reliability	Piezoresistive∼strain gauges Buckling∼3D geometry Piezoresistive∼Microchannels Fluidic∼Conductive Inks Piezoresistive∼strain gauges Sponges and Foams∼PEDOT
**Operational Monitoring**	Low Drift High Accuracy High Repeatability High Reliability	Mixed: Piezoresistive∼strain gauges Buckling∼3D Geometry Capacitive Sponges and Foams∼PEDOT

## Abbreviations

The following abbreviations are used in this manuscript:

FSR	Force Sensing Resistors
PDMS	polydimethylsiloxane
DEAP	dielectric electroactive polymers
PEDOT	Poly(3,4-ethylenedioxythiophene)
rGO	reduced graphene oxide
HCI	Human computer interaction
EGaIn	eutectic gallium-indium
